# Continuous Temperature Telemonitoring of Patients with COVID-19 and Other Infectious Diseases Treated in Hospital-at-Home: Viture^®^ System Validation

**DOI:** 10.3390/s24155027

**Published:** 2024-08-03

**Authors:** Laura Sesma-Sánchez, María Ruiz-Castellano, Ainara Romero-Roldán, Laura Álvarez-García, Marta Morrás-Gómez, Idoia Tabar-Liberal, Marta Pulido-Fontes, Belén Salmón-García

**Affiliations:** 1 Vitio® Medical SL., 31192 Tajonar, Navarra, Spain; 2 Hospital at Home Unit, Navarra University Hospital (HUN), 31008 Pamplona, Navarra, Spain; maria.ruiz.castellano@navarra.es (M.R.-C.); laura.alvarez.garcia@navarra.es (L.Á.-G.); marta.morras.gomez@navarra.es (M.M.-G.); idoia.tabar.liberal@navarra.es (I.T.-L.); marta.pulido.fontes@navarra.es (M.P.-F.); belen.salmon.garcia@navarra.es (B.S.-G.); 3 Navarrabiomed, 31008 Pamplona, Navarra, Spain; ainararomero98@gmail.com

**Keywords:** hospital-at-home, telemonitoring, continuous temperature monitoring, remote patient monitoring, detection of fever, infectious diseases

## Abstract

Body temperature must be monitored in patients receiving Hospital-at-Home (HaH) care for COVID-19 and other infectious diseases. Continuous temperature telemonitoring (CTT) detects fever and patient deterioration early, facilitating decision-making. We performed a validation clinical study assessing the safety, comfort, and impact on healthcare practice of Viture^®^, a CTT system, compared with a standard digital axillary thermometer in 208 patients with COVID-19 and other infectious diseases treated in HaH at the Navarra University Hospital (HUN). Overall, 3258 pairs of measurements showed a clinical bias of −0.02 °C with limits of agreement of −0.96/+0.92 °C, a 95% acceptance rate, and a mean absolute deviation of 0.36 (SD 0.30) °C. Viture^®^ detected 3 times more febrile episodes and revealed fever in 50% more patients compared with spot measurements. Febrile episodes were detected 7.23 h (mean) earlier and modified the diagnostic and/or therapeutic approach in 43.2% of patients. Viture^®^ was validated for use in a clinical setting and was more effective in detecting febrile episodes than conventional methods.

## 1. Introduction

Hospital-at-Home (HaH) is defined as a service that provides active treatment by healthcare professionals in the patient’s home for a condition that would otherwise require acute hospital inpatient care, always for a limited time period [1]. This service, which has emerged in response to the increasing demand for acute care hospital beds, helps avoid admissions to hospital wards, while reducing the risk of adverse events associated with time in hospital and the potential benefit of rehabilitation in the home environment [2,3]. Many studies have analyzed HaH programs [1,2,3,4,5,6,7,8,9] and have demonstrated benefits in terms of safety [4,5,6,7], effectiveness [4,5], reduced iatrogenic complications [5], improved functional recovery [2], and quality of life [8]. Furthermore, the level of patient satisfaction is generally high compared with inpatient care [1,5,6].

Many infectious processes can be followed and treated at home in a safe and effective manner [6,7], including COVID-19 infection [9]. To exploit its full potential, HaH should adapt and take advantage of new opportunities, including technological or medical advances [10]. Remote patient monitoring is associated with improved clinical outcomes and cost-effectiveness of care in chronic diseases, such as chronic heart failure and chronic obstructive pulmonary disease [11,12]. In acute processes, such as infectious diseases, scientific evidence was limited [13] until the arrival of the COVID-19 pandemic, during which its usefulness was also demonstrated [14].

Continuous monitoring of hospitalized patients treated in general medical settings improves outcomes [15], so, plausibly, enhanced monitoring of patients treated in non-ICU settings may be helpful [16]. Continuous telemonitoring of vital signs can have a positive impact on the safety of measurements [17], can lead to earlier detection of patient deterioration [18], and can effectively prioritize patient care.

Studies have already shown the advantages of continuous temperature telemonitoring (CTT) both at hospital and at home [19,20]. Several wearable temperature systems are already available and suitable for use in both environments [19,21,22,23,24,25,26]. They differ mainly in the site of placement (armpit [19,22,23,25], chest [21,24], upper arm [21], or eardrum [26]), the application method (patch-based [19,21,22,23,24,25], band-based [21], or other [26]), whether they are reusable [21,23,25,26] or disposable [19,22,24], the type of battery (replaceable or rechargeable [21,23,26] or not [19,22,24]), battery life (a week or less [19,21,22,24,26] or longer than a month [23,25]), and data transmission technology (Bluetooth Low Energy (BLE) [19,21,23,24,25,26] or NFC [22]). There are also wearables that are worn on the wrist [27] or finger [28], but they measure skin temperature, which fluctuates significantly with the external temperature.

The Viture^®^ CTT system (Vitio^®^, Tajonar, Spain), based on BLE and worn under the armpit, is a very suitable option for monitoring body temperature in patients with COVID-19 and other infectious diseases, as it is comfortable, easy to use, band-based, and reusable with a replaceable battery that lasts a minimum of one year.

The aim of this study was to validate Viture^®^, evaluating the level of agreement with a standard commercially available digital axillary thermometer (Morris MT-401). The study also sought to evaluate the safety and comfort of the system and to evaluate the impact that the introduction of Viture^®^ has on the healthcare practice of an HaH unit. Furthermore, the advantages of Viture^®^ compared with the standard method were evaluated.

## 2. Materials and Methods

### 2.1. Study Design

This was a validation clinical study performed in the HaH unit of the Navarra University Hospital (HUN) [29].

Assuming a confidence level of 95%, a standard deviation of the measurement difference between both measurement methods of 0.35, and an analysis approach using the Bland–Altman method [30], 180 patients (with an average follow-up of 5 days) are needed to detect as significant, with a power of 80%, a discrepancy between measurement techniques greater than clinically relevant, assuming a priori a predetermined clinical agreement interval delta of 0.75. The assumed standard deviation of 0.35 was calculated in a previous pilot project in nursing homes.

### 2.2. Inclusion and Ethics

For this clinical study, we included 208 patients admitted to HaH for COVID-19 or other infectious diseases between the months of February and May 2021 for a maximum of 9 days. The inclusion criteria for participation were as follows:

- To be greater than 18 years old;
- To be able to legally give informed consent;
- To have no limitations to the use of the Viture^®^ device due to physical reasons or interference with other devices.

The criteria for excluding data from the analysis were as follows:

- Monitoring for less than 2 days;
- Less than 4 valid control spot temperature measurements (STMs).

A valid control STM was considered a temperature measured with the reference thermometer that had a simultaneous (within 1 min) Viture^®^ temperature measurement (VTM).

### 2.3. Description of the Viture^®^ System

**Figure 1:**
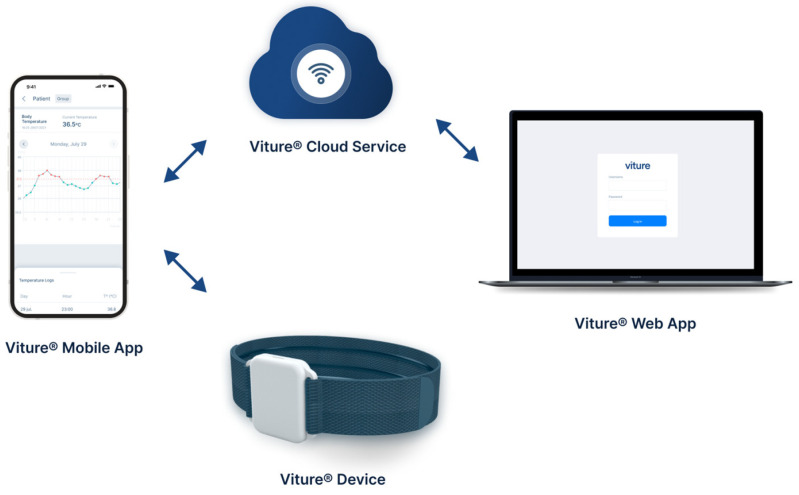
Viture^®^ system developed by Vitio^®^. Continuous temperature telemonitoring system composed of four elements: Viture^®^ device, Viture^®^ mobile app, Viture^®^ web app, and Viture^®^ cloud service.

Viture^®^ (Figure 1), developed by Vitio^®^, is a 24-h CTT system, certified as a Class IIa medical device according to MDR 2017/745 and implemented following the HL7 FHIR healthcare standard protocol for information exchange. It is composed of four elements: the Viture^®^ device, the Viture^®^ mobile app, the Viture^®^ web app, and the Viture^®^ cloud service. The Viture^®^ device (Figure 1) is an easy-to-use, reusable wearable that consists of a comfortable armband with a sensor element that is placed on the user’s arm, under the armpit, which captures axillary temperature at a sampling period of 15 s and transmits the data in real time via BLE. The operating temperature range of the sensor element is [−40, 85] °C; however, the clinical measurement range is [34, 43] °C as the typical range for axillary body temperature. The data emitted by the device are captured by the Viture^®^ mobile app running on a smartphone within its coverage range. The smartphone sends all the collected information to the Viture^®^ cloud servers via the mobile network. In addition, the Viture^®^ device has a flash memory in which the temperature data are stored to prevent data loss and a replaceable battery that lasts a minimum of one year. Once in the cloud, the data can be consulted both from the Viture^®^ mobile app and web app. Additionally, the system sends notifications to the Viture^®^ app when a predefined axillary temperature threshold (37.5 °C) is exceeded.

### 2.4. Protocol

On the first day of the study, the patients were given a study kit containing the following:

- A Viture® device;
- A digital reference thermometer for control STMs (Morris MT-401);
- A smartphone with the Viture® mobile app installed;
- A device user manual in quick guide format;
- A home data collection notebook (HDCN).

The HaH team placed the Viture^®^ device on the patient and trained the patient and/or caregiver. They were asked to complete control STMs between 3 and 6 times a day on the same arm where the Viture^®^ device was placed and record the measuring data in the HDCN.

If the device was removed or the patient experienced discomfort, they were asked to report the incident in the HDCN. Mild incidents were considered skin alterations, such as redness and chafing, and severe incidents were considered hematomas and ulcers.

The HaH team on duty collected all Viture^®^ reports, registering the date, time, and follow-up for each febrile episode. The patient and/or caregiver could be contacted to request a control STM.

### 2.5. Data Collection and Analysis

The statistical analysis was performed with the statistical software R 4.0.5 and the library SimplyAgree [30], and the fever analysis was carried out with Python 3.10. The control STMs were transcribed from the HDCN to .csv files and the VTMs were exported from the Viture^®^ web app into .csv files. For each control STM, the simultaneous (within 1 min) VTM was collected to form 3258 pairs of measurements. From these pairs of control STM and VTM data, only figures equal to or above 35 °C were considered for the statistical analysis. For the analysis of febrile episodes, all the control STMs and Viture^®^ raw data (more than 9 million VTMs) were used.

### 2.6. Statistical Analysis

A statistical analysis of agreement between the digital reference thermometer (Gold-standard) and Viture^®^ was carried out. The non-parametric Wilcoxon signed rank test [31] was used to evaluate the average agreement between the two methods and the Bland–Altman method was used to evaluate the agreement between individual measurements. Both analyses were validated with a 95% confidence interval [32].

### 2.7. Analysis of Febrile Episodes

To explore the true potential of Viture^®^, we performed an analysis of the febrile episodes experienced by patients during the clinical study. Febrile episodes were characterized by their maximum temperature and duration. To assess the impact of Viture^®^ notifications, the data registered on the follow-up of febrile episodes were analyzed. When using the reference thermometer, a single spot measurement of 37.5 °C or higher was considered a febrile episode. When using Viture^®^, a febrile episode was defined as an axillary temperature equal to or above 37 °C that reached 37.5 °C or higher at some point. The episode ended when the temperature dropped below 37 °C and did not rise again to 37 °C after 30 min. The Viture^®^ system’s sensitivity and specificity to detect febrile episodes were calculated.

The mean lead time of Viture^®^ was estimated as the mean of the time differences between the patient’s first febrile episode detected by both methods. Note that this mean lead time was underestimated, as the HaH team could request a control STM after a Viture^®^ notification and the time interval between the febrile episode’s detection and the time when the HaH team was informed was not taken into consideration.

## 3. Results

### 3.1. Data Filtering

A total of 208 patients were monitored during the study period. Of these, 28 were excluded from the study for the following reasons: 18 did not meet the inclusion criteria (minimum days monitored (8) and sufficient control data registered (10)), 3 did not follow the measurement protocol, 3 had insufficient Viture^®^ data (data download errors (2) and the smartphone was off (1)), and 2 were drop-outs, resulting in 180 patients.

During the clinical study, 3258 pairs of control STM and VTM data were obtained and, of these, 993 (30.5%) were discarded for the following reasons: 66 did not have Viture^®^ data available (data download errors and the smartphone was off) and, in 927 pairs, at least one of the temperature values was below 35 °C (in 172 pairs, both values were below 35°C; in 99 pairs, only spot measurements had temperature values below 35°C; and in 656 (20.1%) pairs, only Viture^®^ measurements had temperature values below 35°C). Of the 828 pairs in which VTM measurements were below 35 °C, 747 (22.9%) pairs were discarded due to probable improper armband placement and 81 pairs were discarded because the device was not worn.

### 3.2. Patient Characteristics

**Figure 2:**
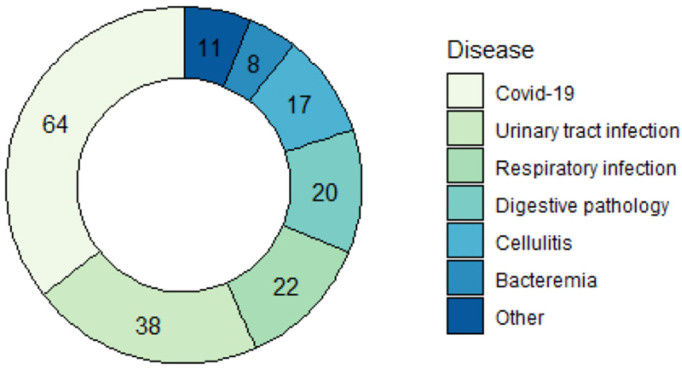
Patient diseases. Of the 180 patients, 35.6% had COVID-19 and 64.4% presented with other infectious diseases, such as a urinary tract infection (21.1%), a respiratory infection (12.2%), a digestive pathology (11.1%), cellulitis (9.4%), bacteremia (4.4%), and other pathologies not indicated above (6.1%).

During the study period, a total of 180 patients were monitored with a mean stay of 5 days. Of these, 56.1% were male and 43.9% were female and the mean age was 63.67 (SD 17.45) years, with a range of [22, 98] years. In terms of referral, 43.3% came from hospital wards, 41.7% from the emergency department, 11.7% from primary care, and 3.3% were outpatients. In terms of disease (Figure 2), 35.6% had COVID-19 and 64.4% suffered from other infectious diseases, such as a urinary tract infection (21.1%), a respiratory infection (12.2%), a digestive pathology (11.1%), cellulitis (9.4%), bacteremia (4.4%), and other pathologies not indicated above (6.1%).

### 3.3. Statistical Analysis

#### 3.3.1. Average Agreement

The mean temperature of the control STMs was 36.09 (SD 0.57) °C with a range of [35, 38.8] °C, and the mean temperature of the VTMs was 36.07 (SD 0.50) °C with a range of [35, 38.7] °C. No statistically significant differences were observed between the averages of each method using the Wilcoxon signed rank test (*p* = 0.0547 > 0.05) with 95% reliability.

#### 3.3.2. Agreement between Individuals

**Figure 3:**
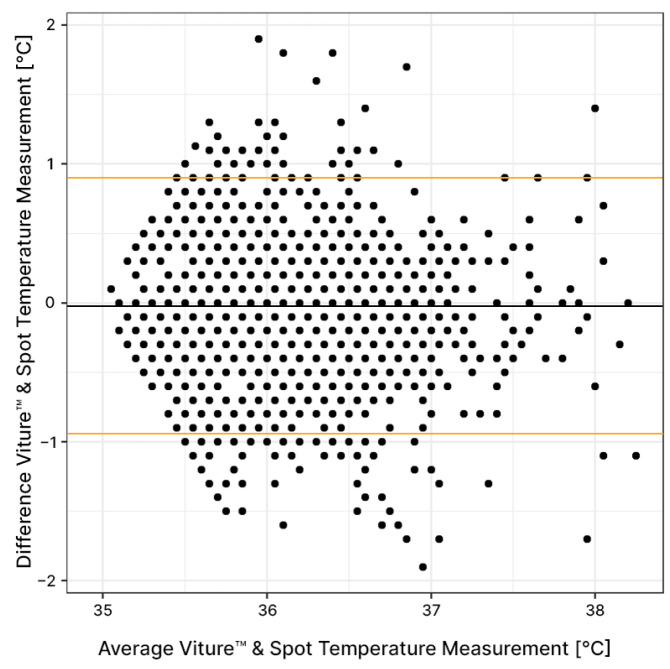
Bland–Altman plot. The difference between Viture^®^ and control spot temperature measurements is displayed versus the arithmetic mean of the temperatures recorded by both methods (black dots). The results show a clinical bias of −0.02 °C (gray line) with limits of agreement of −0.96/+0.92 °C (orange lines) with a 95% acceptance rate.

The Bland–Altman plot presented in Figure 3 shows the difference between control STMs and VTMs versus the arithmetic mean of the temperatures recorded by both methods. The results show a clinical bias of −0.02 °C with limits of agreement of −0.96/+0.92 °C with a 95% acceptance rate. The mean absolute deviation was 0.36 (SD 0.30) °C. Overall, 79.4% of the patients obtained a mean absolute deviation below 0.5 °C and 95% below 0.7 °C.

### 3.4. Analysis of Febrile Episodes

#### 3.4.1. Reference Thermometer Spot Measurements

**Figure 4:**
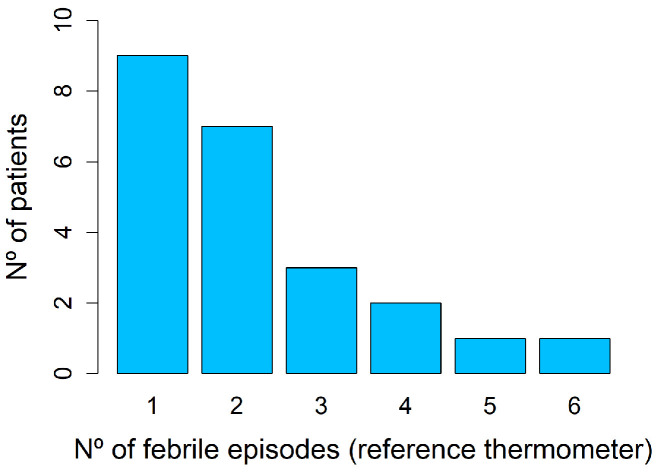
Histogram that illustrates the distribution of patients experiencing a varying number of febrile episodes according to the reference thermometer. In total, 51 febrile episodes corresponding to 23 patients were detected with spot temperature measurements.

A total of 51 febrile episodes were detected with the reference thermometer, corresponding to 23 patients (12.8%). Of these, 56.5% had COVID-19 and 43.5% had other infectious diseases. Figure 4 shows the number of febrile episodes detected per patient.

#### 3.4.2. Comparison between Spot and Viture^®^ Measurements

**Figure 5:**
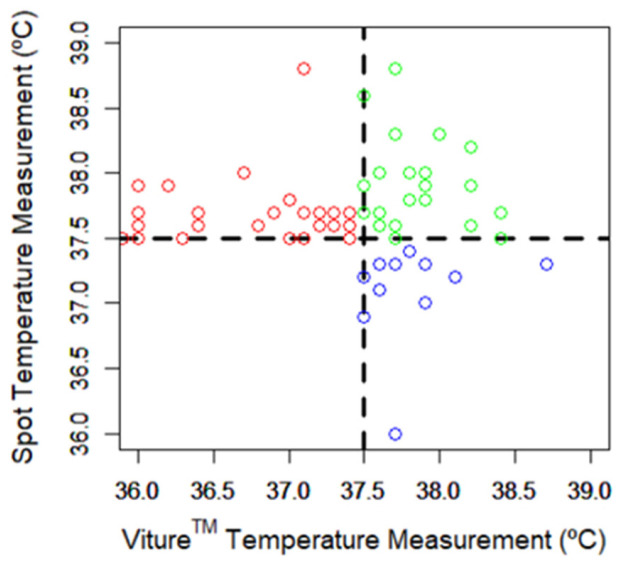
Scatter plot with 64 pairs of control spot temperature measurements and Viture^®^ temperature measurements where at least one of the temperature values was equal to or above 37.5 °C (green, 22 both values; blue, 13 only Viture^®^ measurements; red, 29 only spot measurements).

Figure 5 shows a scatter plot of the 64 pairs of control STM and VTM data where at least one of the temperature values was equal to or above 37.5 °C (green, 22 both values; blue, 13 only Viture^®^ measurements; red, 29 only spot measurements). Of the 51 febrile episodes detected with spot measurements, 22 were simultaneously detected by Viture^®^, 26 cases generated a Viture^®^ notification within ±2 h, and 2 cases generated a Viture^®^ notification within ±12 h. Only in one case, where the control STM was 37.5 °C, did Viture^®^ fail to trigger a notification because it only reached a maximum of 37.4 °C. Viture^®^ reported 48 (94.1%) of the febrile episodes detected by the reference thermometer and in 22 (95.7%) of the 23 patients. Therefore, the sensitivity and specificity of Viture^®^ in detecting febrile episodes were 94.1% and 99.6%, respectively.

Furthermore, Viture^®^ detected three times more febrile episodes and revealed fever in 50% more patients compared with spot measurements. Viture^®^ notifications modified the diagnosis and/or therapeutic approach in 43.2% of the patients with febrile episodes, including unscheduled home visits, the ordering of new diagnostic tests, the identification of infections, and hospital admission due to worsening conditions. Viture^®^ detected 77.3% of the first febrile episode of 22 patients earlier than with spot measurements with a mean lead time of 7.23 h and a range of [0.5, 28.8] h. In the five cases where the spot measurements were detected earlier than Viture^®^, the mean lead time was −3.52 h with a range of [−12, −0.2] h.

#### 3.4.3. Viture^®^ Continuous Temperature Measurements

**Figure 6:**
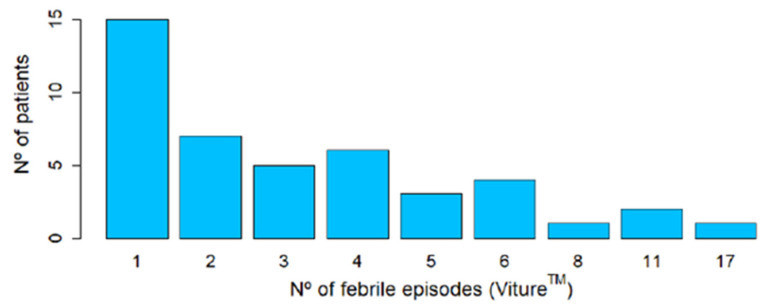
Histogram that illustrates the distribution of patients experiencing a varying number of febrile episodes according to Viture^®^. In total, 154 febrile episodes were detected with continuous temperature measurements, corresponding to 44 patients.

**Figure 7:**
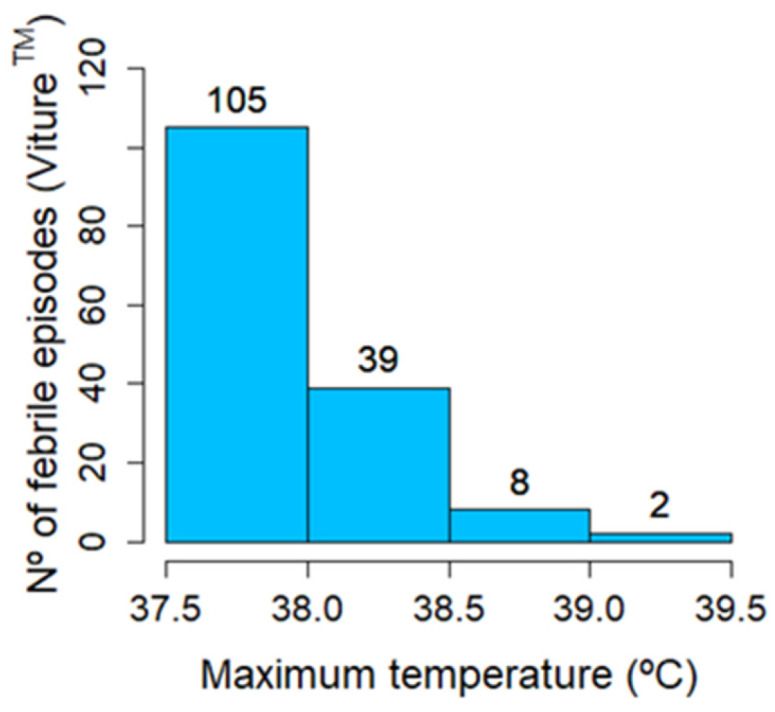
Maximum temperature detected per febrile episode. Most febrile episodes detected had axillary temperatures below 38 °C.

**Figure 8:**
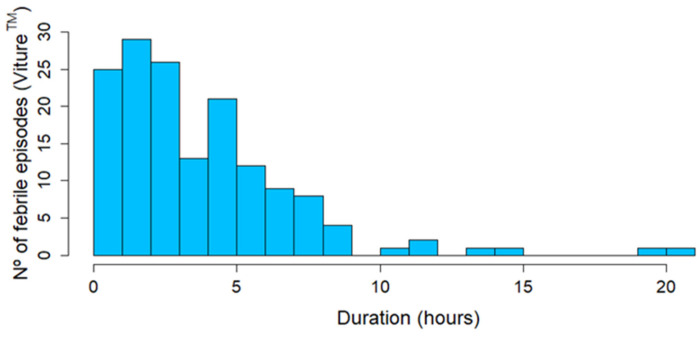
Time duration in hours per febrile episode. Most febrile episodes detected lasted up to 9 h.

With Viture^®^, a total of 154 febrile episodes were detected, corresponding to 44 patients (24.4%). Of these, 45.5% had COVID-19 and 64.5% had other infectious diseases. Figure 6, Figure 7 and Figure 8 show the number of febrile episodes detected per patient, the maximum temperature detected, and the time duration in hours per febrile episode, respectively. Most febrile episodes had axillary temperatures below 38 °C and lasted up to 9 h.

### 3.5. Incidents

During the study, 12 patients suffered an incident while using the Viture^®^ device. Of these, seven patients suffered mild incidents and two suffered severe incidents. The total number of incidents was 15, since some patients suffered more than 1 incident.

Eight patients suffered a skin alteration (three reddening, four chafing, one bruise, and one small sore). Regarding negative sensations, three patients experienced excessive pressure and three patients experienced stinging on three occasions.

## 4. Discussion

Body temperature is a key vital sign in the follow-up of patients that are admitted to HaH with COVID-19 [18,33] and other infectious diseases [34]. In these patients, the detection of a febrile episode may indicate a poor clinical evolution, a lack of a response to treatment, and/or the appearance of new complications. Currently, temperature is mainly monitored using spot measurements [33,35]. CTT can lead to the earlier detection of fever and febrile episodes that otherwise would be missed by the current standard of care, resulting in the earlier detection of patient deterioration [25,36] and early decision-making by the HaH team.

Viture^®^ is a very suitable alternative to continuously monitor the body temperature of patients with COVID-19 and other infectious diseases. It measures axillary temperature, which is already widely used in HaH and other hospital settings, and can be attached with a comfortable armband that does not interfere with the patient’s daily activity. Other locations, such as the chest, upper arm, or eardrum, are not as comfortable, not as easy to attach the device to, or require a patch. Patch-based systems are not as convenient for elderly people with frail skin or a susceptibility to contact dermatitis [37]. They require periodical patch replacement, and they generate waste. Battery life is a critical issue, and a minimum of 14 days is necessary to allow for continuous, uninterrupted monitoring [20]. The fact that Viture^®^ is band-based, is reusable, and has a replaceable battery with a minimum battery life of one year, far longer than other continuous wearable thermometers, makes it a very sustainable solution. This clinical study has validated the use of Viture^®^ in a clinical setting as a substitute for a standard axillary thermometer. The use of Viture^®^ is additionally more effective in detecting febrile episodes thanks to its continuous temperature monitoring. Viture^®^ can detect the frequent episodes that occur at night when patients are sleeping and in the intervals between spot measurements. This real-time telemonitoring of febrile episodes makes it possible to anticipate healthcare actions. This can be especially useful for managing infectious disease outbreaks, as it allows for early detection and reduces hospital admissions, generating a great benefit for public health. Furthermore, based on the collection notebook and the feedback from the HaH team, the patients found the Viture^®^ system easy to use and comfortable. Since they did not have to do anything with it, they even forgot they were wearing it. The patients also reported that they felt reassured to know that a professional was monitoring their health remotely. Although increased patient anxiety is considered a challenge of remote patient monitoring [38], no signals of anxiety were perceived during the study. The acute disease condition of the patients in the study may influence whether they consider such continuous monitoring to be positive and feel better cared for.

Adherence to the Viture^®^ device was generally good. Only two patients (1.0%) dropped out of the study, and the rest wore the device most of the time. Of the 3258 pairs of control STM and VTM data obtained, only 81 (2.5%) were discarded because the device was not worn. Patients sometimes forgot to put the device back on when they took it off to take a shower or they did not wear it the first night because they removed it to sleep. Some patients had it temporarily removed due to discomfort. We had to exclude the data from eight patients because they were monitored for less than two days. Unfortunately, they could not continue with the monitorization because they were admitted to the emergency room or the hospital ward and their stay in HaH ended.

Although the Viture^®^ system has proven to be highly suitable for body temperature monitoring, there remains room for improvement. During the study, some patients suffered skin alterations and negative sensations. Furthermore, sometimes the device moved or was not placed correctly (resulting in improper readings below 35 °C). A total of 22.9% of the pairs of data were discarded because they were below 35 °C. There were also some technical issues with data transmission, such as download errors, the smartphone running out of battery, and no mobile data available. Of all the pairs of data, 2% were removed for this reason. These problems were reduced by disabling the smartphone Wi-Fi to avoid Bluetooth interference with data downloads and by emphasizing that patients keep their smartphones charged or even always plugged in.

However, we should consider that, during the study, the Viture^®^ device was a prototype version and that there are patients in HaH who are elderly and have sensitive skin; therefore, some skin irritation was to be expected. Based on this experience, the use of Viture^®^ may be enhanced by the redesign of the armband and more effective training for clinicians that will help patients to prevent it from slipping off, improve the user experience, and avoid skin redness or chafing. This has been considered for the next Viture^®^ version. It is crucial to be aware that Viture^®^ is a continuous system, and patients may move a lot during the day. While it is true that some Viture^®^ temperature measurements may be less than 35 degrees, accurate measurements can be ensured over longer periods of time.

The study was carried out in the most challenging scenario in an uncontrolled environment and the study patient characteristics are sufficiently distinct to be confident that the system works for a wide range of ages and infectious diseases. These results are encouraging and easily transferable to other diseases. There are many diseases in which temperature monitoring is useful [39,40], and it is likely that technologies such as Viture^®^ will soon become part of the standard of care and be integrated into the day-to-day operation of units such as HaH and other setups. The temperature threshold for notifications can be preset based on the specific disease and adjusted for each individual patient. Furthermore, continuous measurements open up new opportunities for fever prediction and personalized patient outcomes.

We have observed that the use of Viture^®^ has a positive impact on the healthcare practice of HaH; however, more specific studies are required to confirm the clinical and economic advantages of continuous temperature monitoring. It would also be worth evaluating the system in other hospitals and locations to verify that the results are comparable in different settings. Furthermore, other features to be implemented that would facilitate the work and decision-making of the HaH team would be a more sophisticated reporting system and the integration of data into the hospital information systems. As Viture^®^ was implemented following the HL7 FHIR healthcare standard protocol for information exchange, it can be easily integrated into different healthcare systems. The inclusion of other variables, such as heart rate and blood pressure, may also help in clinical decision-making.

## 5. Conclusions

Viture^®^ has been validated for use in a clinical setting and has proven to be more effective in detecting febrile episodes than conventional methods. It offers, in general, detection of more febrile episodes and in an earlier fashion. In addition, it is easy to use, comfortable, reusable, and has a replaceable battery with a minimum battery life of one year, far longer than other continuous wearable thermometers. This makes Viture^®^ easily integrable into clinical practice in HaH units and other medical settings. Continuous temperature monitoring is a tool that can lead to the earlier detection of patient deterioration and facilitate early decision-making and effectively prioritize patient care.

## Data Availability

The authors confirm that the data supporting the findings of this study are available within the article.
